# Patient and Technical Factors Associated with Difficult Arterial Access and Ultrasound Use in the Operating Room

**DOI:** 10.3390/children11010021

**Published:** 2023-12-24

**Authors:** Frank M. Yanko, Adovich Rivera, Eric C. Cheon, John D. Mitchell, Heather A. Ballard

**Affiliations:** 1 Feinberg School of Medicine, Northwestern University Feinberg School of Medicine, Chicago, IL 60611, USA; frank.yanko@northwestern.edu (F.M.Y.); echeon@luriechildrens.org (E.C.C.); 2 Ann & Robert H. Lurie Children’s Hospital of Chicago, Chicago, IL 60611, USA; 3 Henry Ford Health, Detroit, MI 48202, USA; jmitch28@hfhs.org

**Keywords:** catheterization, catheter, arterial, pediatric, retrospective studies, risk factors, ultrasonography

## Abstract

Arterial catheterization enables continuous hemodynamic monitoring but has been shown to cause severe complications, especially when multiple attempts are required. The aim of this study was to explore what factors were associated with multiple attempts and ultrasound use in the operating room. We performed a retrospective analysis of patients who had arterial catheters inserted at a tertiary care children’s hospital from January 2018 to March 2022, identifying clinical factors that were associated with both outcomes. A total of 3946 successful arterial catheter insertions were included. Multivariable analysis showed multiple attempts were associated with noncardiac surgery: pediatric (OR: 1.79, 95% CI: 1.30–2.51), neurologic (OR: 2.63, 95% CI: 1.89–3.57), orthopedic (OR: 3.23, 95% CI: 2.27–4.55), and non-radial artery placement (OR: 5.00, 95% CI: 3.33–7.14) (all *p* < 0.001). Multivariable analysis showed ultrasound use was associated with neonates (OR: 9.6, 95% CI: 4.1–22.5), infants (OR: 6.98, 95% CI: 4.67–10.42), toddlers (OR: 6.10, 95% CI: 3.8–9.8), and children (OR: 2.0, 95% CI: 1.7–2.5) compared to teenagers, with cardiac surgery being relative to other specialties—pediatric (OR: 0.48, 95% CI: 0.3–0.7), neurologic (OR: 0.27, 95% CI: 0.18–0.40), and orthopedic (OR: 0.38, 95% CI: 0.25–0.58) (all *p* < 0.001). In our exploratory analysis, increased odds of first-attempt arterial catheter insertion success were associated with cardiac surgery, palpation technique, and radial artery placement. Younger patient age category, ASA III and IV status, cardiac surgery, and anesthesiologist placement were associated with increased odds of ultrasound use.

## 1. Introduction

Arterial catheterization is a frequently used medical intervention that enables continuous hemodynamic monitoring, blood sampling, and arterial blood gas analysis. An estimated 8 million arterial catheters are placed per year in the United States [1]. Despite its routine use, vascular, infectious, neurologic, embolic, and thrombotic complications have been described [1,2,3,4,5,6,7,8,9,10,11,12]. In adults, major complications such as permanent limb ischemia and sepsis have an incidence under 1% [1]. However, rates of complications in pediatric populations may be higher, as studies have quoted less than 1% to 10.3% for thrombosis, embolism, and infectious complications and up to 59% for arterial line malfunctions [2,9,12]. Multiple placement attempts have been found to be an important risk factor for these complications [2,6].

The number of placement attempts and the difficulty of cannulation are influenced by patient, procedure, and technical factors, but the literature is limited. Younger patient age and female sex have been associated with multiple attempts [1,12,13,14]. Both cardiac surgery and higher ASA status may also be associated with difficult arterial access because these variables are associated with the use of vasopressors, the need for longer indwelling catheters, and arterial catheter insertion complications [3,9]. Brachial and radial artery cannulation, ultrasound use, and smaller catheter size may decrease complication rates and number of attempts in pediatric and adult arterial catheter placement [3,7,8,12,14,15,16,17,18].

Among technical factors, ultrasound use has been shown to increase success and decrease complications [16,19,20]. However, it is unknown what patient and clinical factors are associated with the use of ultrasound for arterial line insertion. Therefore, we conducted an exploratory analysis on which patient and technical factors were associated with multiple attempts and ultrasound use in the operating room.

## 2. Materials and Methods

### 2.1. Study Design and Setting

We performed a retrospective analysis of electronic health record data of patients who underwent arterial catheter insertion in the operating room at the Ann and Robert H. Lurie Children’s Hospital of Chicago from January 2018 to March 2022. The Ann and Robert H. Lurie Children’s Hospital of Chicago Institutional Review Board (IRB) approved this study (LCH IRB 2019-2860) in June 2019 under chairperson Jennifer Rubin. These data were collected as part of a study evaluating vascular access insertion outcomes after an educational intervention [21]. The requirement for written informed consent was waived by the IRB as a retrospective study. This study followed the Strengthening the Reporting of Observational Studies in Epidemiology (STROBE) guidelines (www.strobe-statement.org accessed on 20 May 2019). 

### 2.2. Study Population and Data Collection

We included all patients who underwent successful arterial catheter insertion in the operating room during the study period. Data were collected through a query from the electronic medical records of Epic (Verona, WI, USA). For each procedure, we extracted variables related to the outcomes, patient demographic and clinical factors, and technical factors. At the study institution, it is routine practice to document this information for every arterial catheter insertion in the operating room.

### 2.3. Outcome

We studied two outcomes of interest: (1) multiple attempts for arterial catheter insertion and (2) ultrasound use in the successful attempt (versus not using ultrasound for the successful attempt). Number and success of attempts were based on the arterial catheter procedure note. Clinicians were instructed to count an arterial catheter insertion attempt as any time the skin was punctured by a needle. Attempts were defined as new penetrations of the skin with the needle, and multiple attempts were defined as more than one skin puncture required for successful placement, as defined in the literature previously [18,22]. 

### 2.4. Patient Demographic and Clinical Factors

Patient demographic information included age group, sex, race/ethnicity, body mass index (BMI) percentile, and comorbidities (inpatient status, American Society of Anesthesiologists Physical Status, congenital heart disease, prematurity, renal disease, trisomy 21, and surgical specialty). Age group was categorized by neonate (0–<1 month), infant (1–<12 months), toddler (12–<36 months), child (36–<144 months), and teenager (144–<216 months). Race/ethnicity was self-reported into categories of Black non-Hispanic, White Hispanic, and White non-Hispanic. All other racial/ethnicity categories were excluded due to containing less than 5% of the sample population. 

Body mass index (BMI) was calculated using the extracted weight and height from the electronic medical record. BMI percentile was then calculated by the Centers for Disease Control and Prevention BMI calculator: underweight <5th percentile, healthy weight 5th–<85th percentile, overweight 85th–<95th percentile, and obese 95th percentile or greater. As there are no BMI norms for children less than 2 years old, BMI percentiles could not be calculated for this age group.

Comorbidities were extracted from the EMR using International Classification of Disease 9 and 10 Clinical Modification (ICD-9-CM and ICD-10-CM) codes associated with the patient’s record. The ICD-9-CM and ICD-10-CM codes were as follows: congenital heart disease (745–747, Q20–28), prematurity (765, P05, P07), renal disease (580–589, 753, N00–08, P96, Q61–64, N10–16, N17–19), and trisomy 21(758, Q90) [23]. Surgical specialty was determined by the proceduralist listed and categorized as cardiac surgery/cardiology, pediatric surgery, neurosurgery, orthopedic surgery, and other specialties (dental, gastroenterology, medical imaging, ophthalmology, otolaryngology, plastic, and urology). Other specialties were grouped together due to the low numbers of arterial lines inserted (<5%). 

### 2.5. Technical Factors

The following information was extracted from the arterial catheter procedure note: date of service, number of attempts, ultrasound use, practitioner placing arterial catheter successfully (attending anesthesiologist, certified registered nurse anesthetist (CRNA), or trainee (resident, fellow, student nurse anesthetist)), and artery location (radial, ulnar, and other). The artery locations in the other category (posterior tibial, femoral, dorsalis pedis) were pooled due to small numbers (<5%). At the study institution, there are no set protocols on which patients receive ultrasound guidance for arterial catheter insertion. Ultrasound use is at the discretion of the attending anesthesiologist.

### 2.6. Statistical Methods

We calculated descriptive statistics for patient and technical factors stratified according to first-attempt success versus multiple attempts. Appropriate tests of group differences (*t*-test for normally distributed continuous variables and Chi-square for categorical variables) were used to test for significant differences between outcome groups. 

We then used multivariable logistic regression model to identify predictors associated with the outcomes. All outcome models were adjusted for age category, sex, race/ethnicity, patient comorbidities, and technical variables (year, practitioner type, and artery location). For the multiple-attempt success outcome, we also included the use of ultrasound as a predictor. These variables were selected based on literature published on arterial catheter complications [1,2,3,4,9,14,22]. Catheter size was initially included as a candidate predictor variable, but we found it to be collinear with age group, creating issues in model calculation. We, therefore, opted to remove catheter size and retain age group in our modeling. 

Since BMI was not available for those less than 2 years old, we performed a sensitivity analysis where BMI category was included as predictor, but those <2 years old were excluded from the analytical sample. This led to a total of 4 multivariable models (2 outcomes, 2 model versions per outcome). Missing data were addressed using multiple imputations. A sensitivity analysis was also conducted using complete case analysis. All analyses were conducted in R3.4 (RStudio, Vienna, Austria).

## 3. Results

### 3.1. Unadjusted Analysis

Data from 4114 arterial catheter insertions were extracted from the EMR. Seventy-five catheter insertions were excluded due to being placed outside of the OR, and 93 (2.3%) were excluded for not having the outcome of interest. A total of 3946 successful arterial catheter insertions were analyzed in this study. A total of 725 (18%) placements were missing an artery location, 364 (13%) were missing the BMI category, and 118 (3%) were missing ASA physical status. The rest of the predictors were missing 1% or less data. 

Eight hundred sixty-eight arterial catheters required multiple attempts for placement (22.0%). Characteristics of patients with and without first-attempt success are listed in Table 1. Ultrasound was used successfully 84.0% of the time in the first-attempt success group and 83.9% of the time in the multiple-attempt group.

### 3.2. Multivariable Analysis

In the multivariable analysis shown in Table 2, patient and technical factors associated with multiple attempts included the following: noncardiac surgery: pediatric surgery (OR: 1.79, 95% CI: 1.30–2.51, *p* < 0.001), neurosurgery (OR: 2.63, 95% CI: 1.89–3.57, *p* < 0.001), orthopedic surgery (OR: 3.23, 95% CI: 2.27–4.55), earlier study year (OR: 0.92, 95% CI: 0.85–0.99, *p*: 0.022), ultrasound use relative to palpation technique (OR: 1.33, 95% CI: 1.05–1.67, *p* = 0.019), and non-radial artery placement (femoral, brachial, dorsalis pedis) (OR: 5.00, 95% CI: 3.33–7.14, *p* < 0.001). In children requiring multiple attempts at arterial line placement, the attending anesthesiologist was more likely to be successful: CRNA (OR: 0.27, 95% CI: 0.19–0.41, *p* < 0.001) and trainee (OR: 0.34, 95% CI: 0.28–0.41, *p* < 0.001). Patient factors of age category, sex, race/ethnicity, inpatient status, ASA status, and comorbidities (congenital heart disease, prematurity, renal disease, and trisomy 21) were not found to be associated with multiple attempts.

The results of the multivariable model for ultrasound use are shown in Table 3. Patient and technical factors associated with increased use of ultrasound included all age groups relative to teenager, neonate (OR: 9.6, 95% CI: 4.1–22.5, *p* < 0.001), infant (OR: 6.98, 95% CI: 4.67–10.42, *p* < 0.001), toddler (OR: 6.10, 95% CI: 3.8–9.8, *p* < 0.001), and child (OR: 2.0, 95% CI: 1.7–2.5, *p* < 0.001); ASA status 3 (OR: 1.62, 95% CI: 1.11–2.38, *p* = 0.013) and ASA 4 (OR: 1.87, 95% CI: 1.11–3.16, *p* = 0.019) relative to ASA 1; cardiac surgery relative to all other specialties; pediatric surgery (OR: 0.48, 95% CI: 0.3–0.7, *p* < 0.001); neurosurgery (OR:0.27, 95% CI: 0.18–0.40, *p* < 0.001); orthopedic surgery (OR: 0.38, 95% CI: 0.25–0.58, *p* < 0.001); time (year, OR: 1.56, 95% CI: 1.43–1.71, *p* < 0.001); and anesthesiologist relative to CRNA (OR: 0.48, 95% CI: 0.33–0.69, *p* < 0.001). Sex, race/ethnicity, inpatient status, patient comorbidities (congenital heart disease, prematurity, renal disease, and trisomy 21), and artery location were not associated with the use of ultrasound. 

A sensitivity analysis using the BMI category and excluding ages less than 2 years old yielded similar results for both first-attempt success and ultrasound use. BMI category was not associated with first-attempt success or ultrasound use. A sensitivity analysis using complete case analysis also showed similar results, except that outpatient class was associated with multiple attempts (OR: 1.26, 95% CI: 1.00–1.58, *p* = 0.046) and prematurity was inversely associated with ultrasound use (OR: 0.45, 95% CI: 0.22–0.95, *p* = 0.029).

## 4. Discussion

In this retrospective analysis of intraoperative arterial catheter insertions, we found that noncardiac surgery and non-radial artery location were associated with multiple attempts. In patients that required multiple attempts, the attending anesthesiologist and ultrasound use were more likely to be successful. In addition, patients who had their arterial catheters inserted with ultrasound were more likely to be younger (ASA 3 and 4 statuses), undergoing cardiac surgery, and having the attending anesthesiologist perform placement. Our study adds to the literature by delineating potential patient and technical factors that may be associated with multiple attempts, which can be further tested through confirmatory studies. 

In contrast to what has been published in the literature [8,16,24,25,26,27,28,29], we found that ultrasound use and the attending anesthesiologist inserting the arterial catheter were associated with multiple attempts. In our institution, ultrasound is often used for arterial line placement (84% overall) but is also used as a rescue method when palpation techniques have failed. In comparison to our results, studies that showed ultrasound increased first-attempt success randomized patients to either catheter insertion with palpation and ultrasound techniques [14,22,30,31,32]. Due to the limitations of our EMR query, only the successful arterial catheter insertion attempts are documented. Therefore, we do not know which practitioner performed previous attempts or whether ultrasound was used for those attempts. Anecdotally, attending anesthesiologists will attempt arterial catheter insertion after the trainee or CRNA has attempted it once or twice. It is, therefore, possible that non-attending anesthesiologists and palpation techniques were used for these failed attempts, but these details were not captured in our dataset. At our institution, ultrasound is used after failed attempts to ensure the target artery remains suitable and there is no appreciable vasospasm or hematoma [33,34].

In addition, we also found that cardiac surgery was associated with higher first-attempt success and ultrasound use. In comparison, Nuttall and colleagues found that cardiac surgery was associated with arterial catheter insertion complications [3]. We believe that our contrasting findings may be due to the high volume of arterial catheter insertion procedures that these practitioners perform on a routine basis. The high volume of arterial catheter insertions may be associated with increased practitioner skill but also with more complications (though not necessarily an overall higher incidence) [35]. Cuper and colleagues also found that practitioners with a high volume of experience (IV nurses) were associated with fewer intravenous catheter insertion attempts [36].

Interestingly, both ultrasound use and first-attempt success were significantly associated with later study years. We believe that these results reflect the increased adoption of ultrasound for pediatric vascular access and may be a translational effect of an educational intervention [37]. As part of a quality improvement initiative, all attending anesthesiologists, CRNAs, and trainees participated in a simulation-based mastery learning curriculum in ultrasound-guided intravenous catheter insertion in 2018–2019 [21]. This intervention was shown to increase first-attempt success rates and ultrasound use for ultrasound-guided intravenous catheter insertion [38]. Since the needling technique is similar for both arterial and intravenous catheters, we believe this intervention may have also been associated with improved use of ultrasound and first-attempt success for arterial catheter insertion.

In contrast to the literature, we found that there was no association between multiple attempts and female sex or younger age groups. A potential reason cited in these studies is that multiple attempts are required because it is more difficult to cannulate small-diameter arteries [13,14,15,39]. Though we did not find any difference in multiple attempts for younger age groups, we did find that younger and sicker (ASA III and IV) patients were more likely to have ultrasound used for successful arterial catheter insertion. At our institution, there is no protocol for ultrasound use; however, many practitioners will use ultrasound pre-emptively for anticipated difficult arterial catheter insertions. By “seeing the vessels”, ultrasound may be able to mitigate the increased difficulty experienced when attempting to cannulate innately small arteries or arteries with increased vascular tone (i.e., sepsis or vasopressor administration). 

We also found that there was no difference in multiple attempts and self-reported race/ethnicity. This finding contrasts the previous literature that darker skin and Black race were associated with higher odds of difficult intravenous access [23,40,41]. A potential reason for this difference is that arterial catheter insertion does not depend on vessel visibility on the skin surface in comparison to landmark IV insertions. In our previous study, we did find that ultrasound was more likely to be used in IV insertions for Black non-Hispanic patients [23]. However, we found no racial or ethnic differences in the use of ultrasound for arterial catheter insertions in our institution, though the use of ultrasound for arterial catheter insertions was high overall (84%). 

This study had several limitations. First, causation cannot be determined due to data source limitations. Specifically, we were unable to investigate if ultrasound was used as a rescue method after palpation techniques because the use of ultrasound was only documented on the successful attempt. Anecdotally, practitioners at our institution may attempt arterial line insertion using palpation techniques and switch to using ultrasound when unsuccessful. In addition, some significant associations may have occurred by chance alone due to the multiple variables analyzed in this exploratory study or due to confounding variables. Second, there may be selection bias regarding which patients were chosen to undergo arterial catheters under ultrasound versus palpation technique. As there are no set protocols for ultrasound use for vascular access, the modality used for placement is at the discretion of the attending anesthesiologist. Third, there is more likely to be missing data due to the retrospective nature of this study. However, the outcomes of interest and most predictor variables were present in greater than 95% of the procedure notes. Fourth, this study was limited to a tertiary care pediatric hospital, so findings may not be generalizable to different care environments. Fifth, patient comorbidities (renal disease, cardiac disease, prematurity, and trisomy 21) were based on ICD9/10-CM billing codes, which brings the risk of misclassification bias [42,43]. Sixth, the self-reported nature of the data may lead to misclassification bias, of which patients were labeled as having difficult arterial catheter insertions. Finally, only information on the successful arterial catheter insertion attempt was documented in the procedure note. Therefore, we were unable to determine what practitioner performed previous attempts, what arteries were attempted, and whether ultrasound was used for those previous attempts. 

## 5. Conclusions

In our exploratory analysis, the increased odds of multiple arterial catheter insertion attempts were associated with noncardiac surgery, ultrasound use, and non-radial artery placement. The attending anesthesiologist was more likely to be successful if multiple attempts were required for success. Younger patient age category, ASA III and IV status, cardiac surgery, and anesthesiologist placement were associated with increased odds of ultrasound use. Plans for future studies include focusing on collecting prospective data to determine whether the implementation of difficult vascular access algorithms can lead to improved patient care for children undergoing arterial catheter insertion.

## Figures and Tables

**Table 1:** Baseline patient and technical characteristics of 3946 arterial catheter insertions in the operating room.

Characteristic	First Attempt *(n* = 3078) *n* (%)	Multiple Attempts (*n* = 868) *n* (%)	*p*-Value
Age in years, mean (SD)	6.92 (6.10)	7.07 (6.23)	0.524
Sex			0.01
Female	1483 (48.2)	462 (53.2)	
Male	1595 (51.8)	406 (46.8)	
Race			0.516
White non-Hispanic	1459 (47.4)	388 (44.7)	
Black non-Hispanic	457 (14.8)	133 (15.3)	
White Hispanic	958 (31.1)	290 (33.4)	
Other	204 (6.6)	57 (6.6)	
Body Mass Index Category			0.724
Underweight (<5th percentile)	215 (11.2)	64 (11.9)	
Healthy Weight (5–84th percentile)	1156 (60.4)	325 (60.6)	
Overweight (85–94th percentile)	251 (13.1)	61 (11.4)	
Obese (>95th percentile)	293 (15.3)	86 (16.0)	
Patient Class			0.168
Outpatient	1779 (57.8)	525 (60.5)	
Inpatient	1299 (42.2)	343 (39.5)	
ASA Physical Status			<0.001
I	136 (4.6)	50 (5.9)	
II	522 (17.5)	205 (24.2)	
III	1800 (60.4)	481 (56.9)	
IV	524 (17.6)	110 (13.0)	
Congenital Heart Dx	1311 (42.6)	293 (33.8)	<0.001
Prematurity	147 (4.8)	51 (5.9)	0.221
Renal Disease	495 (16.1)	120 (13.8)	0.117
Trisomy 21	121 (3.9)	41 (4.7)	0.346
Surgical Subspecialty			<0.001
Cardiac Surgery/Cardiology	1248 (40.6)	254 (29.3)	
Dental	0 (0.0)	8 (0.3)	
Gastroenterology	3 (0.3)	4 (0.1)	
Medical Imaging	33 (3.8)	143 (4.7)	
Neurological Surgery	172 (19.9)	444 (14.5)	
Ophthalmology	0 (0.0)	4 (0.1)	
Otolaryngology	20 (2.3)	79 (2.6)	
Orthopedic Surgery	207 (23.9)	444 (14.5)	
Pediatric Surgery	121 (14.0)	431 (14.0)	
Plastic Surgery	26 (3.0)	73 (2.4)	
Pulmonology	0 (0.0)	1 (0.0)	
Transplant Surgery	22 (2.5)	161 (5.2)	
Urology	8 (0.9)	32 (1.0)	
Year			<0.001
2018	460 (14.9)	190 (21.9)	
2019	709 (23.0)	224 (25.8)	
2020	810 (26.3)	188 (21.7)	
2021	871 (28.3)	203 (23.4)	
2022	228 (7.4)	63 (7.3)	
Ultrasound Use	2584 (84.0)	728 (83.9)	0.997
Practitioner Type			<0.001
Attending Anesthesiologist	942 (30.8)	436 (51.4)	
CRNA	295 (9.7)	33 (3.9)	
Trainee	1819 (59.5)	379 (44.7)	
Artery Location			<0.001
Axillary	3 (0.1)	3 (0.4)	
Brachial	4 (0.2)	1 (0.1)	
Dorsalis Pedis	7 (0.3)	16 (2.3)	
Femoral	22 (0.9)	18 (2.5)	
Posterior Tibial	18 (0.7)	26 (3.7)	
Radial	2225 (88.6)	600 (84.5)	
Ulnar	232 (9.2)	46 (6.5)	

ASA, American Society of Anesthesiologists; BMI, body mass index; CRNA, certified registered nurse anesthetist; SD, standard deviation.

**Table 2:** Multivariable logistic regression to evaluate patient and technical factors associated with multiple attempts at arterial catheter insertion in the operating room.

Characteristic	Odds Ratio	95% CI	*p*-Value
Age Category			
Teenager	Reference		
Child	0.91	0.74–1.14	0.407
Toddler	0.72	0.52–1.00	0.053
Infant	1.3	0.99–1.69	0.056
Neonate	1.2	0.79–1.82	0.39
Sex			
Female	Reference		
Male	0.93	0.79–1.10	0.375
Race			
White non-Hispanic	Reference		
Black non-Hispanic	1.05	0.75–1.47	0.768
White Hispanic	1.06	0.83–1.35	0.632
Other	1.14	0.94–1.37	0.186
Patient Class			
Outpatient	Reference		
Inpatient	0.88	0.72–1.08	0.219
ASA Physical Status			
I	Reference		
II	1.3	0.88–1.92	0.179
III	1.05	0.72–1.54	0.795
IV	0.81	0.51–1.28	0.361
Congenital Heart Dx	0.79	0.61–1.02	0.07
Prematurity	1.2	0.81–1.79	0.361
Renal Disease	0.91	0.71–1.17	0.473
Trisomy 21	1.33	0.88–2.04	0.174
Surgical Subspecialty			
Cardiac Surgery/Cardiology	Reference		
Pediatric Surgery	1.79	1.30–2.51	<0.001
Neurosurgery	2.63	1.89–3.57	<0.001
Orthopedic Surgery	3.22	2.27–4.55	<0.001
Other	1.27	0.95–1.67	0.101
Time (Year)	0.92	0.85–0.99	0.022
Ultrasound Use	1.33	1.05–1.67	0.019
Practitioner Type			
Attending Anesthesiologist	Reference		
CRNA	0.27	0.19–0.41	<0.001
Trainee	0.34	0.28–0.41	<0.001
Artery Location			
Radial	Reference		
Ulnar	1.32	0.91–1.89	0.15
Other	5	3.33–7.14	<0.001

ASA, American Society of Anesthesiologists; CRNA, certified registered nurse anesthetist; Ref, reference.

**Table 3:** Multivariable logistic regression to evaluate patient and technical factors associated with ultrasound use at arterial catheter insertion in the operating room.

Characteristic	Odds Ratio	95% CI	*p*-Value
Age Category			
Teenager			
Child			
Toddler	Reference		
Infant	2.04		
Neonate	6.1	1.65–2.52	<0.001
Sex	6.98	3.81–9.75	<0.001
Female	9.6	4.67–10.42	<0.001
Male		4.1–22.48	<0.001
Race	Reference		
White non-Hispanic	0.93		
Black non-Hispanic		0.77–1.13	0.461
White Hispanic	Reference		
Other	0.85		
Patient Class	0.93	0.56–1.28	0.428
Outpatient	0.96	0.70–1.23	0.603
Inpatient		0.78–1.20	0.739
ASA Physical Status	Reference		
I	1.09		
II		0.86–1.39	0.455
III	Reference		
IV	1.3		
Congenital Heart Dx	1.62	0.88–1.91	0.189
Prematurity	1.87	1.11–2.38	0.013
Renal Disease	1.19	1.11–3.16	0.019
Trisomy 21	0.56	0.87–1.62	0.282
Surgical Subspecialty	1.02	0.28–1.11	0.097
Cardiac Surgery/Cardiology	1.35	0.75–1.38	0.92
Neurological Surgery		0.62–2.94	0.452
Orthopedic Surgery	Reference		
Pediatric Surgery	0.48		
Other	0.27	0.31–0.73	<0.001
Time (Year)	0.38	0.18–0.40	<0.001
Practitioner Type	0.47	0.25–0.58	<0.001
Attending Anesthesiologist	1.56	0.32–0.68	<0.001
CRNA		1.43–1.71	<0.001
Trainee	Reference		
Artery Location	0.48		
Radial	1.05	0.33–0.69	<0.001
Ulnar		0.84–1.31	0.664
Other	Reference		
	1.55		
	0.74	0.74–3.22	0.235
		0.39–1.44	0.377

ASA, American Society of Anesthesiologists; CRNA, certified registered nurse anesthetist; Ref, reference.

## Data Availability

The data that support the findings of this study are available from Ann and Robert H. Lurie Children’s Hospital of Chicago. Restrictions apply to the availability of these data, which were used under license for this study. Data are available from the authors with the permission of Ann and Robert H. Lurie Children’s Hospital of Chicago.
